# Isolation of tumour stem-like cells from benign tumours

**DOI:** 10.1038/sj.bjc.6605142

**Published:** 2009-06-30

**Authors:** Q Xu, X Yuan, P Tunici, G Liu, X Fan, M Xu, J Hu, J Y Hwang, D L Farkas, K L Black, J S Yu

**Affiliations:** 1 Department of Neurosurgery, Cedars-Sinai Medical Center, Los Angeles, CA, USA; 2 Department of Pathology and Laboratory Medicine, Cedars-Sinai Medical Center, Los Angeles, CA, USA; 3 Department of Surgery, Cedars-Sinai Medical Center, Los Angeles, CA, USA

**Keywords:** pituitary adenomas, tumour stem cells, growth hormone, benign tumours

## Abstract

**Background:**

Cancerous stem-like cells (CSCs) have been implicated as cancer-initiating cells in a range of malignant tumours. Diverse genetic programs regulate CSC behaviours, and CSCs from glioblastoma patients are qualitatively distinct from each other. The intrinsic connection between the presence of CSCs and malignancy is unclear. We set out to test whether tumour stem-like cells can be identified from benign tumours.

**Methods:**

Tumour sphere cultures were derived from hormone-positive and -negative pituitary adenomas. Characterisation of tumour stem-like cells *in vitro* was performed using self-renewal assays, stem cell-associated marker expression analysis, differentiation, and stimulated hormone production assays. The tumour-initiating capability of these tumour stem-like cells was tested in serial brain tumour transplantation experiments using SCID mice.

**Results:**

In this study, we isolated sphere-forming, self-renewable, and multipotent stem-like cells from pituitary adenomas, which are benign tumours. We found that pituitary adenoma stem-like cells (PASCs), compared with their differentiated daughter cells, expressed increased levels of stem cell-associated gene products, antiapoptotic proteins, and pituitary progenitor cell markers. Similar to CSCs isolated from glioblastomas, PASCs are more resistant to chemotherapeutics than their differentiated daughter cells. Furthermore, differentiated PASCs responded to stimulation with hypothalamic hormones and produced corresponding pituitary hormones that are reflective of the phenotypes of the primary pituitary tumours. Finally, we demonstrated that PASCs are pituitary tumour-initiating cells in serial transplantation animal experiments.

**Conclusion:**

This study for the first time indicates that stem-like cells are present in benign tumours. The conclusions from this study may have applications to understanding pituitary tumour biology and therapies, as well as implications for the notion of tumour-initiating cells in general.

Pituitary adenomas are benign brain tumours, but they can cause significant morbidity due to overexpression of pituitary hormones (Levy, 2004) or neurological symptoms such as visual loss and headache (Greenman and Melmed, 1996; Melmed, 2003). Pituitary adenomas account for about 10% of diagnosed intracranial neoplasms, typically during early adulthood, yet they are found in 6–24% of adult autopsies (Laws and Jane, 2001). The classification of these tumours is usually based on the types of pituitary hormones they produce, such as prolactin (PRL), growth hormone (GH), adrenocorticotropic hormone (ACTH), luteinising hormone (LH), follicle-stimulating hormone (FSH), thyroid-stimulating hormone (TSH), and others (Levy, 2004). Prolactinomas are the most common type; they cause amenorrhoea, galactorrhoea, and infertility in women and hypogonadism in men. Somatotrophic adenomas secrete an excess of GH, and cause gigantism in children and acromegaly in adults. ACTH-secreting adenomas produce Cushing's disease, whereas gonadrotophic (secreting LH and FSH) and thyrotropic adenomas are rare, with the latter causing hyperthyroidism (Levy, 2004). Some pituitary adenomas does not secrete hormones and they are classified as null-cell adenomas, with their diagnosis based on visual difficulties arising from the compression of the optic nerve (Greenman and Melmed, 1996; Melmed, 2003). Pituitary adenomas are also classified based on the size of the tumours: macroadenomas are greater than 10 mm in diameter and microadenomas have a diameter of less than 10 mm (Shomali and Katznelson, 2002).

Important insights into pituitary adenoma tumorigenesis may come from studies of pituitary development and cell differentiation. Pituitary embryogensis is orchestrated by a combination of intrinsic and extrinsic signals including basic fibroblast growth factors (bFGF), Wnt, sonic hedgehog, and Notch signals (Scully and Rosenfeld, 2002; Heaney and Melmed, 2004; Zhu *et al*, 2007). Pituitary stem cells may also exist in adult tissues. It had been reported that a side population from mouse anterior pituitary is enriched in stem-like cells that express stem/progenitor cell markers, but not differentiated cell markers, and can grow in floating spheres in culture (Chen *et al*, 2005). The side population also contains some folliculostellate cells (FSCs), the bFGF-producing stem-like cells. However, nestin-positive cells isolated from rat pituitary displayed features of sphere forming, motility, and proliferation, but are distinct from FSCs (Krylyshkina *et al*, 2005). The identification of adult pituitary stem cells remains unclear.

Recent discoveries of cancer stem-like cells (CSCs) as cancer-initiating cells in malignant cancers, such as gliobastomas (Yuan *et al*, 2004; Xu *et al*, 2008), led to other important questions. Is the presence of CSCs correlated to malignancy? Do stem-like cells exist in benign tumours, such as pituitary adenomas? Targeted expression of human pituitary tumour-transforming gene in mouse pituitary stem/progenitor cells led to plurihormonal hyperplasia and adenomas (Abbud *et al*, 2005), suggesting possibly a transformed stem cell origin in the tumours. In addition, an FSC-like cell line was developed from a pituitary adenoma (Danila *et al*, 2000). However, whether these cells share stem-like cell characteristics, and whether these cells can initiate pituitary adenomas *in vivo* remains unknown.

In this study, we report the isolation of cloned single cells from both hormone-producing and hormone-null pituitary adenomas with the ability to self-renew in sphere culture. These pituitary adenoma-derived stem-like cells (PASCs) express a range of stem cell-associated markers and lineage-specific stem/progenitor cell markers. When differentiated, these cells downregulate stem cell-associated genes and produce multiple pituitary hormones in response to hypothalamic hormone stimulation. Finally, when transplanted into NOD/SCID mice, these sphere-forming cells generate intracranial tumours that can be serially transplanted to recapitulate the human tumour.

## Materials and methods

### Primary culture of pituitary tumour stem-like cells

Tumour stem-like neurospheres were prepared as reported previously (Yuan *et al*, 2004). Briefly, tumour samples from eight pituitary adenoma patients were collected within 30 min after the surgical resection as approved by the Institutional Review Board at Cedars-Sinai Medical Center. Tissues were washed three times in PBS 1 × and dissected in a dissection medium containing catalase, deferoxamine, *N*-acetyl cysteine, and superoxide dismutase. After digestion in trypsin for 10 min at 37°C, tumour tissues were triturated by passing them in a tissue sieve and after recovering the cells by passing in a 70 *μ*m cell strainer. Cells were then plated at the density of 1 × 10e5 cells per ml in a medium containing DMEM/F12 (1 : 1; Gibco, Carlsbad, CA, USA), B27 1 × (Gibco), penicillin/streptomycin (200 U ml^−1^; Gibco), fungizone (250 ng ml^−1^), EGF (20 ng ml^−1^; Peprotech, Rocky Hill, NJ, USA), and bFGF (20 ng ml^−1^; Peprotech). For the differentiation of pituitary tumour stem cells, single-cell suspensions were switched to the differentiation culture medium (DMEM/F12 medium with 2 mM glutamine, 15% horse serum, and 2.5% fetal bovine serum). After the pituitary tumour cells attached to the culture dish, these cells were allowed to grow in the differentiation medium for 7–10 days.

### Subsphere formation assays

Subsphere formation assays were performed in 96-well culture plates as described before (Yuan *et al*, 2004). Briefly, the spheres were mechanically dissociated into single cells and diluted into culture medium. The suspended cells were seeded into 96-well plates with the dilution that would result in one cell per well. The single–cell-containing wells were identified by checking the culture wells 2 h after the cell-seeding process. After 2 weeks of culture with the medium refreshed every 3 days, the culture wells were observed and the subsphere containing wells were counted. This subsphere assay was done with different passages of the sphere cells and experiments were repeated once.

### Immunofluorescence staining of tumour cell spheres and differentiated daughter cells

Tumour cell spheres were harvested and whole spheres were fixed in 4% paraformaldehyde and stained with antibodies against nestin (1 : 100; Chemicon, Temecula, CA, USA), CD133 (1 : 200; Abcam, Cambridge, MA, USA), and GH (1 : 2000; Chemicon). For staining of differentiated cells, cells were fixed in 4% paraformaldehyde, followed by several washing with PBS and permeabilised in 0.1% Triton X-100. After blocking with 10% goat serum in PBS, cells were then incubated with primary antibodies: GFAP (1 : 200; Dako, Carpinteria, CA, USA), *β*-tubulin III (1 : 400; Covance, Berkeley, CA, USA), and S100 (1 : 200; Chemicon). After incubation with FITC or Texas Red-conjugated secondary antibodies (1 : 300; Jackson ImmunoResearch, West Grove, PA, USA), slides were counterstained with a mounting medium containing DAPI (Vector Laboratories, Burlingame, CA, USA) before examination by fluorescence microscopy.

### RNA isolation and cDNA synthesis

Total RNA was extracted from fresh tumour tissue, PASCs, and their daughter cells using an RNA4PCR kit (Amibion, Austin, TX, USA) according to the manufacturer's protocol. For cDNA synthesis, 1 *μ*g total RNA was reverse transcribed into cDNA using Oligo dT primer and iScript cDNA synthesis kit reverse transcriptase. cDNA was stored at −20°C for subsequent PCR analysis.

### Real-time quantitative RT-PCR

Gene expression was quantified by real-time quantitative RT-PCR using QuantiTect SYRB Green dye (Qiagen, Valencia, CA, USA). DNA amplification was carried out using Icycler (Bio-Rad, Hercules, CA, USA). All the primer sets were provided by Qiagen as shown in Supplementary Table 1. The relative quantities of target gene mRNA against an internal control, GAPDH, was calculated using a Δ*C*_T_ method and an amplification plot with fluorescence signal *vs* cycle number was drawn. The difference (Δ*C*_T_) between the mean values in the duplicated samples of target genes and those of GAPDH were calculated and the relative quantified value (RQV) was expressed as 2^−Δ*C*_*T*_^.

The relative expression of each gene presented in each clone was compared between PASC and differentiated cells.

### Chemotherapeutic resistance analysis

Tumour stem-like cells and adherent daughter cells were also tested for resistance to chemotherapeutic treatment. Cells were treated with 50 *μ*M carboplatin and 12.5 *μ*M VP16 for 48 h and the toxicity was assessed by WST-1 proliferation assay (Roche, Indianapolis, IN, USA).

### Stimulated hormone production

Pituitary hormone production by PASC1, PASC2, and their differentiated daughter cells were determined using ELISA. Cells were cultured with or without 1 × 10^−7^ M GH-releasing factor (GHRF), 2 × 10^−7^ M PRL-releasing peptide (PRP), 1 × 10^−7^ M LH-releasing hormone (LHRH), 1 × 10^−7^ M thyrotropin-releasing hormone (TRH), and 1 × 10^−7^ M corticotropin-releasing hormone (CRH) for 24 h. The secreted hormones (GH, PRL, LH, TSH, FSH, and ACTH) in the conditioned media were determined using ELISA kits (Anogen, Mississauga, Ontario, Canada). The results are the average±standard deviation of three independent experiments. For immunofluorescence staining of human GH and FSH expressed in differentiated PASCs, cells were fixed and stained with antibodies specific to human hormones at 24 h after hypothalamic hormone stimulation. Nuclei were detected with DAPI staining.

### Implantation of pituitary adenoma stem-like cells in mice

NOD/SCID mice (6–8 weeks old; Jackson Laboratory, Bar Harbor, Maine, USA) were anesthetised with i.p. ketamine and medetomidine, and stereotactically implanted with pituitary adenoma sphere cells (PASC1–4 and human glioblastoma U87 cells, 10 000 per mouse) in the right forebrains. At 3 and 6 months after implantation, two animals from each of the above implanted mice were killed, followed by intracardiac perfusion–fixation with 4% paraformaldehyde. Immunostaining of the brain sections was performed with human-specific antibody against human cell nuclei (1 : 100; Chemicon) and also with antibody against human-specific growth hormone (1 : 2000; Chemicon). Alternatively, the implanted mice were killed at 12 weeks. The mouse forebrains around the implantation site were harvested under a dissection microscope and the dissected samples were homogenised. A human growth hormone-specific ELISA kit was used to detect the levels of human GH in the homogenised tissues. For serial transplantation, fresh human tumour cells harvested from the brains of mice that survived 6 months after implantation were cultured under the same conditions as described above and expanded as spheres. The same number of pituitary adenoma cells was implanted into mice following the same procedure as described above. At 3 months after implantation, animals were killed and the brains were subject to histological analysis for secondary tumour formation. All animals used were treated in strict accordance with the Institutional Animal Care and Use Committee guidelines enforced at the Cedars-Sinai Medical Center.

## Results

### Isolation of PASCs and their self-renewal and multipotent capacity

**Figure 1:**
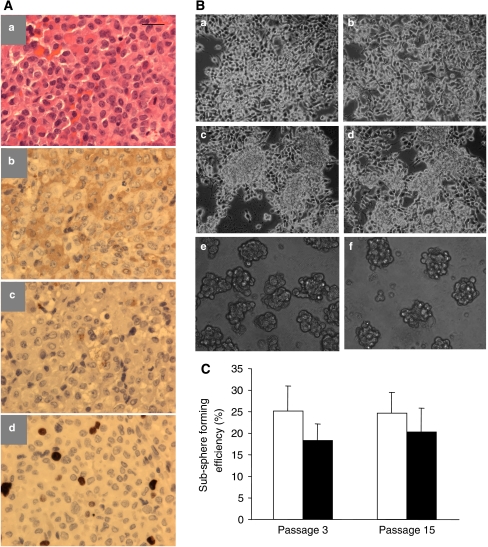
Pituitary adenoma stem-like cells form self-renewable spheres in culture. (**A**) Histological characterisation of a human pituitary somatotroph adenoma: (a) Hematoxylin and eosin; (b) human GH; (c) human PRL; and (d) Ki67 staining. Scale bar, 10 *μ*m. (**B**) Tumour cells from one growth hormone-positive (PA1; a, c, e) and one null-cell (PA2; b, d, f) pituitary adenomas were cultured in serum-free neural stem cell medium. Initially tumour cells grow as monolayers with epithelial cell morphology (a, b). In a week, tumour cells form spheres on top of the monolayers (c, d). Both tumour stem-like cells are continuously propagated as free-floating spheres in culture (e, f). (**C**) Single tumour stem-like cells were cloned from both growth hormone-positive (PASC1) and null cell (PASC2) tumour spheres. There was no significant change in subsphere-forming efficiency of both PASC1 (open bar) and PASC2 (closed bars).

Previously, we had isolated brain tumour stem-like cells from malignant brain tumours, namely, glioblastoma multiforme (Yuan *et al*, 2004; Xu *et al*, 2008). To investigate whether stem-like tumour-initiating cells exist in benign tumours or specifically, in pituitary adenomas, we cultured tumour cells from a number of clinical specimens of pituitary tumours. Detailed characterisation and functional studies were focused on tumour cells from one GH-positive somatotroph tumour and one hormone-negative null-cell macroadenoma. The GH-positive somatotroph tumour was positive in human GH staining and cell proliferation antigen staining, with moderate PRL staining (Figure 1A). Under the culture conditions identical to those for neural stem cells, spheres resembling typical neurospheres readily formed on top of the monolayers of both cells (Figure 1B a–d). The spheres could be propagated and continuously cultured in a free-floating form (Figure 1B e and f). Single cells were cloned from spheres for each tumour and expanded for subsequent studies. To assess their self-renewal capacity, subsphere-forming assays were performed for both cells. At passages 3 and 15, both cells displayed consistent subsphere-forming efficiency (Figure 1C). Therefore, both tumour cells forms self-renewable spheres in culture.

**Figure 2:**
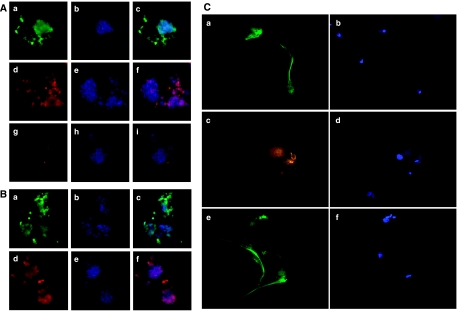
Pituitary adenoma cells express neural stem cell markers and possess multipotential differentiation capability. (**A** and **B**) Immunofluorescence staining of pituitary tumour spheres from PASC1 (**A**) and PASC2 (**B**) with antibodies against nestin (green; **A**-a and **B**-a) and CD133 (red; **A**-d and **B**-d). Nuclei were detected with DAPI staining (blue; **A**-b, e, h and **B**-b, e). Overlaps of antibody and nuclei staining were shown (**A**-c, f, i and **B**-c, f). (**C**) Single-cell suspension from tumour cell spheres was plated and grown in differentiation medium for 7–10 days. Immunofluorescence staining of differentiated pituitary adenoma stem-like cells (PASC1) was performed with antibodies against *β*-tubulin III (a), GFAP (c), and S100 (e). Nuclei were revealed with DAPI staining (b, d, f).

To test whether these sphere-forming tumour cells express cell markers, which are shared by neural stem cells and glioblastoma stem-like cells, both tumour cells were stained with immunofluorescence using antibodies against nestin and CD133. As shown in Figure 2A and B, both tumour cells express neural stem cell markers nestin and CD133. In addition, GH-positive staining was found only in dispersed somatotroph tumour cells, but not in sphere-forming cells (Figure 2A g–i). Thus, sphere-forming pituitary tumour cells express stem/progenitor cell markers, but not differentiated cell markers. Next, sphere-forming tumours were cultured under differentiation conditions for 1 week and analysed for the expression of lineage-specific markers. Upon differentiation, these cells acquired an epithelial morphology resembling original pituitary adenoma cells. Differentiated cells were found to express neural-specific *β*-tubulin III, astroglial protein GFAP, and brain-specific protein S-100 (Figure 2C), suggesting that these cells had multipotent differentiation capacity. Therefore, we have isolated sphere-forming, self-renewable, and multipotent pituitary adenoma stem-like cells (PASCs) from both GH-positive somatotroph tumours and null-cell tumours, PASC1 and PASC2, respectively.

### Expression of stem cell-associated genes by PASCs and chemosensitivity analysis

**Figure 3:**
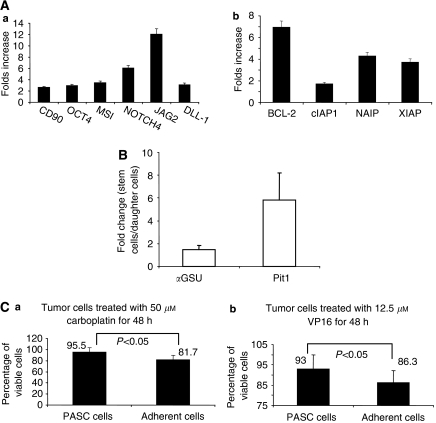
Pituitary adenoma stem-like cells preferentially express stem cell-associated genes and display drug resistance. (**A**) Ratios of expression levels of stem cell-associated genes (a) and antiapoptotic genes (b) in tumour stem-like cells relative to adherent daughter cells. Total RNA was isolated from tumour stem-like cells and their differentiated daughter cells. The mRNA expression levels of each gene were quantified using real-time PCR methods and the ratios were calculated using normalised expression data. (**B**) Ratios of expression levels of pituitary stem cell-associated genes (*α*GSU and Pit1) in tumour stem-like cells relative to adherent daughter cells. (**C**) Chemosensitivity in tumour stem-like cells and their daughter cells. Tumour stem-like cells and daughter cells were treated with carboplatin and VP16 at the indicated concentrations for 48 h. Surviving cells were quantified using WST-1 assays.

To further characterise these benign tumour-derived stem-like cells, we determined the gene expression levels of a series of stem-cell-associated genes in both PASC1 and their daughter cells. CD90, OCT4, Musashi-1 (MSI), NOTCH4, JAG2, and DLL-1 are typically expressed in embryonic or adult stem cells (Kaneko *et al*, 2000; Kabos *et al*, 2002; Nagato *et al*, 2005). We found that expression levels of these stem cell-associated genes were higher in PASC1 than in its differentiated daughter cells (Figure 3A a). Stem cells are generally known to preferentially express antiapoptotic genes, such as BCL-2, cIAP1, NAIP, and XIAP (Jin *et al*, 2008). It was determined that the expression levels of these antiapoptotic genes in PASC1 were one- to sixfolds higher than those in its daughter cells (Figure 3A b). There was no significant difference in the expression levels of multiple drug resistance related genes, such as BCRP1, MDR1, MRP1, and MRP3 (Donnenberg and Donnenberg, 2005; Abbott, 2006), between PASC1 and the daughter cells (data not shown). Next, we determined gene expression levels of some genes important for pituitary development, including Pit-1, *α*GSU, GATA2, and other genes. Pit-1 is a transcription factor that regulates expression of GH and PRL. Pit-1 is expressed in progenitor cells in developing pituitary glands and its expression precedes GH messenger RNA expression in fetal pituitary glands (Scully and Rosenfeld, 2002). GATA2 is a zinc finger transcription factor necessary for differentiation and determination of gonadotrophs and thyrotrophs (Scully and Rosenfeld, 2002). The expression of Pit-1 is over fourfold higher in PASC1 than in the daughter cells (Figure 3B). The expression of *α*GSU is also increased in PASC1 (Figure 3B), whereas GATA2 is similarly expressed in both PASC1 and the daughter cells. No detectable mRNA expression of LH, FSH, TSH, ACTH, NeuroD1 (Oyama *et al*, 2001), Tpit, SF-1 (Barnhart and Mellon, 1994), and PROP1 was found in either PASC1 or the daughter cells (data not shown).

We reported in a previous study that glioblastoma stem-like cells are characterised by their high resistance to chemotherapeutic agents, including carboplatin, etoposide (VP16), and other drugs (Liu *et al*, 2006). To test whether PASC1 shares this property with malignant tumour stem-like cells, we treated PASC1 and its daughter cells with carboplatin and VP16 and performed toxicity assays. As shown in Figure 3C, PASC1 manifested significantly higher resistance than the daughter cells to both chemotherapeutic treatments. Therefore, PASCs express a broad range of stem cell-associated genes and possess chemotherapeutic resistance property.

### PASCs are responsive to hypothalamic hormones and can produce multiple pituitary hormones

**Figure 4:**
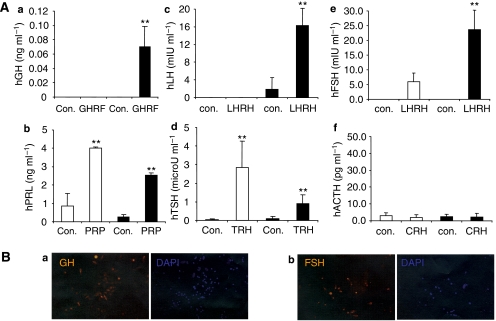
Pituitary adenoma stem-like cells can differentiate into hormone-producing cells. (**A**) Pituitary hormone production by differentiated PASC1 in response to corresponding hypothalamic hormones. PASC1 (open bars) or its differentiated daughter cells (closed bars) were cultured with or without 1 × 10^−7^ M GH-releasing factor (GHRF; a), 2 × 10^−7^ M PRL-releasing peptide (PRP; b), 1 × 10^−7^ M LH-releasing hormone (LHRH; c and e), 1 × 10^−7^ M thyrotropin-releasing hormone (TRH; d), and 1 × 10^−7^ M corticotropin-releasing hormone (CRH; f) for 24 h. The secreted hormones (GH, PRL, LH, TSH, FSH, and ACTH) in the conditioned media were determined using ELISA kits (Anogen). ^**^P<0.01. (**B**) Immunofluorescence staining of GH (a) and FSH (b) expressed in differentiated PASC1 after hypothalamic hormone stimulation. Nuclei were detected with DAPI staining.

One hallmark of differentiated pituitary cells is their responsiveness to hypothalamic hormones and the ensuing production of pituitary hormones. To investigate whether PASCs can be differentiated into hormone-producing cells, both PASC1 and PASC2 were cultured in a differentiation medium for 2 weeks, followed by stimulation of hypothalamic hormones, including GHRF, PRP, LHRH, TRH, and CRH. The conditioned media from PASC1, PASC2, and their respective daughter cells were analysed using ELISA assays for the presence of main pituitary hormones, including GH, PRL, FSH, LH, TSH, and ACTH. As shown in Figure 4A, only cells differentiated from PASC1 responded to GHRF and LHRH and produced GH, FSH, and LH (Figure 4A a,c,e). Interestingly, both PASC1 and its daughter cells responded to PRP and TRH and produced PRL and TSH (Figure 4A b,d). Neither PASC1 nor its daughter cells produced ACTH regardless of stimulation (Figure 4A f). In contrast, cells differentiated from macroadenoma-derived PASC2 manifested different responsiveness to hypothalamic hormone stimulation and hormone production properties. Neither PASC2 nor its daughter cells produced GH, PRL, TSH, or ACTH in the presence or absence of hypothalamic hormone stimulation. However, only undifferentiated PASC2 produced LH, and only differentiated PASC2 produced FSH, in response to LHRH stimulation (Supplementary Figure 1). Therefore, PASCs can be differentiated into functional, hormone-producing cells that respond to hypothalamic hormone stimulation, and their distinct hormone-producing properties reflected the characterisation of the corresponding primary pituitary adenomas.

### PASCs initiated pituitary tumours that are serially transplantable

**Figure 5:**
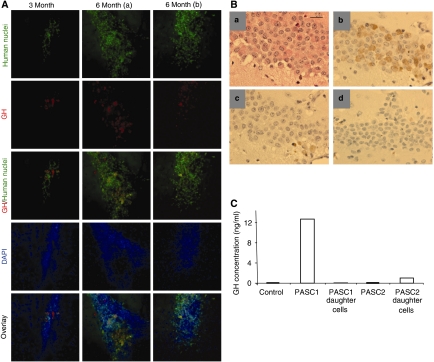
Pituitary adenoma stem-like cells can initiate transplantable, hormone-positive brain tumours. (**A**) Immunofluorescence staining of brain sections from mice implanted with somatotroph pituitary adenoma-derived PASC1 for 3–6 months using antibodies against human-specific nuclei and human GH. Two different samples of 6 months (a and b) were shown with similar results. Human-specific antibody stained cells were visualised by FITC-conjugated secondary antibody (green). The human GH-positive cells were identified by Texas Red-conjugated secondary antibody (red). DAPI was used to identify nuclei (blue). The overlay images were showed as well. (**B**) Histological staining of mouse brain sections at 6 weeks after PASC1 implantation: (a) hematoxylin and eosin; (b) human GH; (c) human PRL; and (d) Ki67 staining. Scale bar, 10 *μ*m. (**C**) Forebrains around the implantation site from the mice at 3 months were harvested under a dissection microscope. The dissected samples were homogenised. A human GH-specific ELISA kit was used to detect the human GH levels in the tissue extract. Tumours derived from glioblastoma (U87) cells were used as control.

We finally studied whether these PASCs are the pituitary tumour-initiating cells. To test whether the PASCs have the ability to form new tumours *in vivo*, PASCs (1 × 10^4^ cells per mouse), their daughter cells (1 × 10^5^ cells per mouse), or glioblastoma cells (1 × 10^4^ cells per mouse, as control) were stereotactically implanted into the right hemisphere of NOD/SCID mice. At 3 months after the intracranial implantation, human-specific cells were identified by immunostaining in the brains of mice that received PASC implantation. However, there was no human-specific cell found within the brains of mice that received the daughter cell implantation (Figure 5A; only results from PASC1 are shown). At 6 months after the intracranial implantation, larger areas of human-specific cell masses were identified in the brains of mice that received PASC implantation compared with that of 3 months after the intracranial tumour spheres implantation (Figure 5A). There was no human-specific cell found within the brains of mice that received daughter cells at 6 month. Furthermore, only brain tumours from PASC1 cells were GH-positive, as indicated by immunostaining of brain sections with a human GH-specific antibody (Figure 5A). Histological staining indicated that PASC1 tumours indeed expressed human GH, as well as moderate human PRL, whereas only a minority of tumour cells were proliferative based on Ki67 staining (Figure 5B), similar to the histology and hormone production features of the primary human tumours (see Figure 1A). To investigate whether the new tumours initiated from PASCs were serially transplantable, the PASC-generated tumour masses within the brain were harvested at 6 months after the intracranial implantation. The harvested tissues were primarily cultured under the same conditions as those used for the isolation of PASCs. Sphere-forming cells were identifiable in the culture and the spheres can be propagated as free-floating spheres in stem cell culture medium. These sphere cells were retransplanted again into the brains of NOD/SCID mice (1 × 10^4^ cells per mouse). At 3 months post-transplantation, the mice were killed and the brain tissues were processed for human-specific cell identification. All three mice with the transplantation were found to contain cell masses that are positive for human-specific nuclei staining, and some cells within the masses were human GH-positive as well (Supplementary Figure 2), suggesting that serially transplanted brain tumours maintain the hormone production property of the primary human tumours and the first round xenografts (see Figure 5A).

To further test whether these PASC cell-initiated brain tumours indeed share the property of the primary pituitary tumours for hormone production, tumours in the mouse forebrains at 12 weeks after implantation were harvested under a dissection microscope. The dissected samples were homogenised and the tissue extract was analysed using a human GH-specific ELISA kit. Significant amount of human GH was detected only in the samples from tumours derived from PASC1, but not from tumours from differentiated PASC1, PASC2, or glioblastoma cells (Figure 5C). Therefore, PASCs can initiate pituitary tumours that recapitulate the phenotypes of human primary tumours, and PASCs are pituitary adenoma-initiating cells.

## Discussion

Cancerous stem-like cells have been implicated as cancer-initiating cells in a range of malignant tumours (Lessard and Sauvageau, 2003; Singh *et al*, 2004; Yuan *et al*, 2004; Bao *et al*, 2006; O'Brien *et al*, 2007; Ricci-Vitiani *et al*, 2007; Malanchi *et al*, 2008; Xu *et al*, 2008). Whether there are any stem-like tumour-initiating cells in benign tumours is unclear. In this study, we isolated sphere-forming, self-renewable, and multipotent stem-like cells from pituitary adenomas, typically benign tumours. We found that PASCs, compared with their differentiated daughter cells, expressed increased levels of stem cell-associated gene products, antiapoptotic proteins, as well as developing pituitary progenitor cell markers. Similar to cancer stem cells isolated from glioblastomas, PASCs are more resistant to chemotherapeutics than their differentiated daughter cells. Furthermore, differentiated PASCs responded to stimulation with hypothalamic hormones and produced corresponding pituitary hormones that are reflective of the phenotypes of the primary pituitary tumours. Finally, we demonstrated that PASCs are pituitary tumour-initiating cells in serial transplantation animal experiments.

The cancer stem cell theory is currently at the centre of broad and extensive investigations with both supporting and qualifying evidences. Recent identification of CSCs from gliomas (Singh *et al*, 2004; Yuan *et al*, 2004; Lee *et al*, 2006), prostate cancers (Collins *et al*, 2005), colon cancers (O'Brien *et al*, 2007; Ricci-Vitiani *et al*, 2007), and breast cancers (Yu *et al*, 2007) suggested that CSCs as cancer-initiating cells may be a general theme in human cancers. Furthermore, it is proposed that CSCs may be responsible for cancer resistance to radiation and chemotherapeutics treatments (Bao *et al*, 2006; Liu *et al*, 2006). However, unlike normal tissue stem cells, CSCs may be heterogeneous (Wicha, 2008; Wright *et al*, 2008; Xu *et al*, 2008), meaning that all CSCs from tumours of the same tissue origin and grade, or even from the same tumour, are not the same. For example, both CD44^+^/CD24^−^ and CD133^+^ cell populations had been identified from the same cancer in a BRCA breast cancer model (Wright *et al*, 2008), suggesting that one initial mechanism may lead to diverse CSCs with different phenotypes. This conclusion may explain the paradoxical findings from a recent study identifying non-CD133^+^ cells as colon cancer-initiating cells (Shmelkov *et al*, 2008), as opposed to results from two other studies (O'Brien *et al*, 2007; Ricci-Vitiani *et al*, 2007). As another example, we have identified both hedgehog signalling-dependent and -independent CSCs from human glioblastomas (Xu *et al*, 2008). For these hedgehog signalling-dependent CSCs, suppression of hedgehog signalling inhibited CSC self-renewal and CSC-initiated brain tumour growth. Hedgehog signalling activity in PTEN-expressing glioblastomas was negatively associated with survival time of the cancer patients. This recent study suggested, on the other hand, that CSCs from different glioblastomas are not equivalent in their underling genetic programs, signalling pathways, and resultant cancer progression, prognosis, and responses to signalling-specific targeted treatments. The qualitative difference among CSCs implies that the mere existence or quantity of CSCs in a tumour may not be a predictor of malignancy or tumour progression. Our current study of isolation of tumour stem-like cells from benign tumours, although generally supporting cancer stem cell hypothesis, further reinforced the postulation that tumour stem-like cells are not associated with malignancy.

Our study is one of the first to isolate and phenotypically and functionally characterise pituitary adenoma stem-like cells. Lineage analysis and transcriptional control studies in developing pituitary gland generated knowledge about pituitary tissue stem cells and their functions (Zhu and Rosenfeld, 2004; Lee *et al*, 2005; Virard *et al*, 2006; Zhu *et al*, 2006). Vankelecom and colleagues isolated murine pituitary cells from side populations that can be clonally expanded and share many stem cell characteristics (Chen *et al*, 2005; Krylyshkina *et al*, 2005; Vankelecom, 2007). Other laboratories also identified pituitary colony-forming cells and their multipotent differentiation capabilities (Lepore *et al*, 2005, 2006; Mogi *et al*, 2005). Although propagating cells or cell lines that share certain stem-like cell markers had been isolated from pituitary tumours (Danila *et al*, 2000; Horvath and Kovacs, 2002; Abbud *et al*, 2005), stringent functional characterisation of pituitary tumour stem-like cells has been scarce. In this study, we phenotypically and functionally characterised PASCs from both hormone-positive and -negative pituitary adenomas. We demonstrated that these PASCs were capable of self-renewal and multipotent differentiation *in vitro* and initiation of serially transplantable pituitary tumours *in vivo*. Importantly, only PASCs from GH-positive somatotroph tumours can initiate tumours that are also GH-positive. Our study conclusively demonstrated the existence of stem-like cells in benign tumours.

Most recently, there has been evidence suggesting that tumour stem-like cells may not be so rare and that the concept may not apply to all types of cancers. It has been shown that a high frequency (10% or higher) of certain lymphomas and leukaemia may initiate new cancers (Kelly *et al*, 2007). Although this finding is interesting, its implications remain unclear as it is based on experimentally engineered murine tumours. In another recent report, Morrison and colleagues discovered that the frequency of melanoma cancer-initiating cells may be as high as one quarter, as long as completely immune deficient mice (NOD/SCID/IL2R*γ*-null mice), instead of widely used NK cell-containing NOD/SCID mice, were used as recipient mice (Quintana *et al*, 2008). It is possible that the cancer stem cell theory may not apply to all types of cancer, such as melanomas. In reality, human cancers develop and progress in patients who possess immune systems of various functionality. To what degree the murine models apply to human cancers remains an open question.

Clearly, more investigations are warranted to further understand the molecular aspects of these PASCs. For instance, what, if any, are the specific molecular markers that distinct PASCs from non-tumour-initiating cells? What molecular mechanisms underlie the self-renewal of PASCs? Is there any relationship between PASCs and pituitary stem cells? What are the defining differences between stem-like cells from benign tumours and from malignant tumours? Future studies in this area will help answer these questions and bring us more knowledge about tumour stem-like cells in most cancers.

In summary, we have isolated self-renewable and multipotent stem-like cells from various pituitary adenomas. We demonstrated that these pituitary tumour stem-like cells, when implanted into immune compromised mice, can initiate transplantable pituitary tumours that resemble the primary tumours. This study for the first time indicated that stem-like cells are present in benign tumours. The conclusions from this study may have applications to understanding pituitary tumours, as well as implications in cancer stem cell theory in general.
