# Supplementary material for: Isolation of tumour stem-like cells from benign tumours
Source: Br J Cancer. 2009 Jun 30;101(2):303–11. doi: 10.1038/sj.bjc.6605142 (PMC2720199; doi:10.1038/sj.bjc.6605142)
Supplement: Supplementary Figure S1 [file 6605142x1.ppt]

## Slide 1
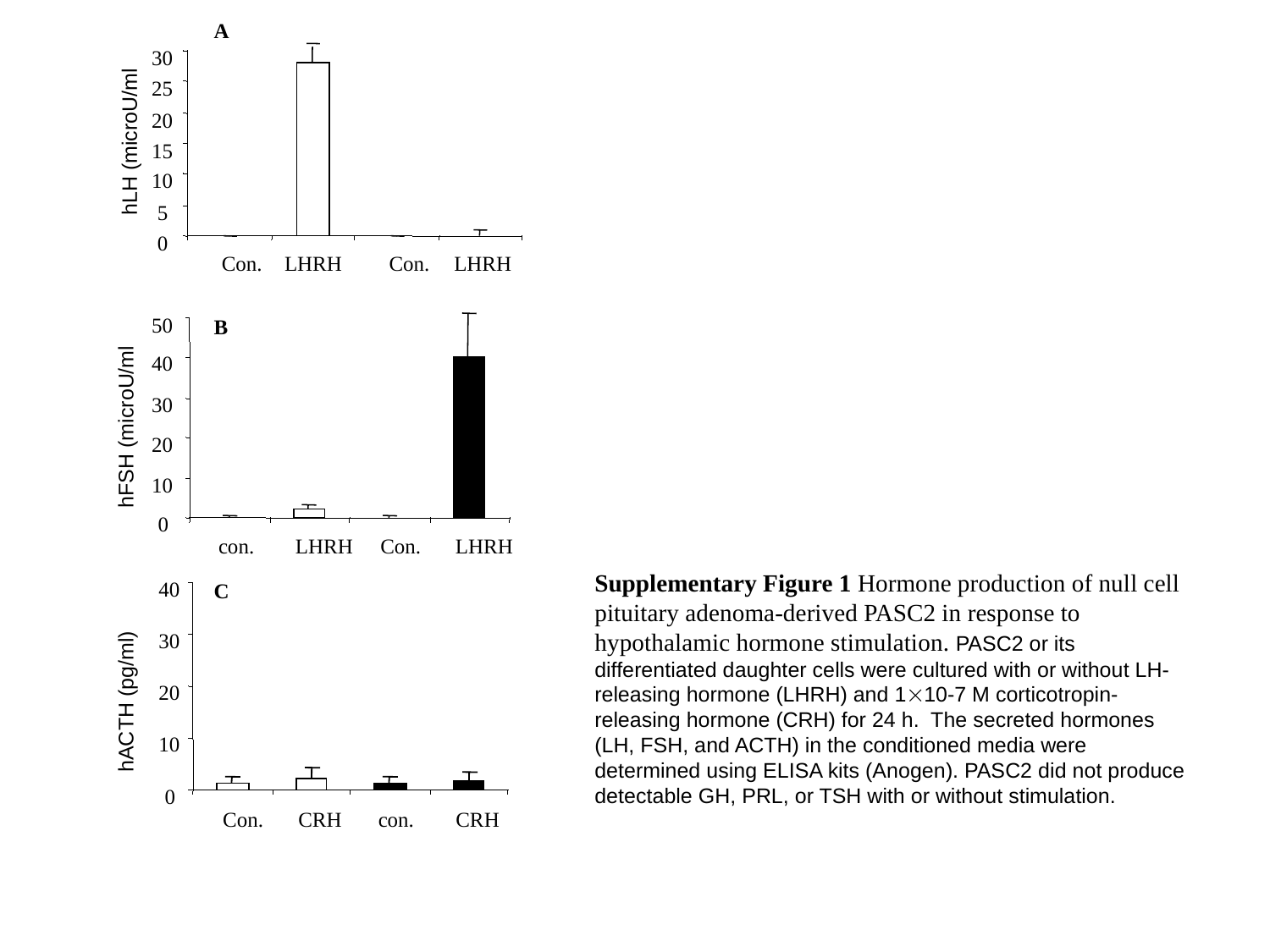

A
30
25
20
hLH (microU/ml
15
10
5
0
Con.
LHRH
Con.
LHRH
B
50
40
30
hFSH (microU/ml
20
10
0
con.
LHRH
Con.
LHRH
Supplementary Figure 1 Hormone production of null cell pituitary adenoma-derived PASC2 in response to hypothalamic hormone stimulation. PASC2 or its differentiated daughter cells were cultured with or without LH-releasing hormone (LHRH) and 1×10^-7^ M corticotropin-releasing hormone (CRH) for 24 h. The secreted hormones (LH, FSH, and ACTH) in the conditioned media were determined using ELISA kits (Anogen). PASC2 did not produce detectable GH, PRL, or TSH with or without stimulation.
C
40
30
hACTH (pg/ml)
20
10
0
Con.
CRH
con.
CRH
