# Supplementary material for: Isolation of tumour stem-like cells from benign tumours
Source: Br J Cancer. 2009 Jun 30;101(2):303–11. doi: 10.1038/sj.bjc.6605142 (PMC2720199; doi:10.1038/sj.bjc.6605142)
Supplement: Supplementary Figure S2 [file 6605142x2.ppt]

## Slide 1
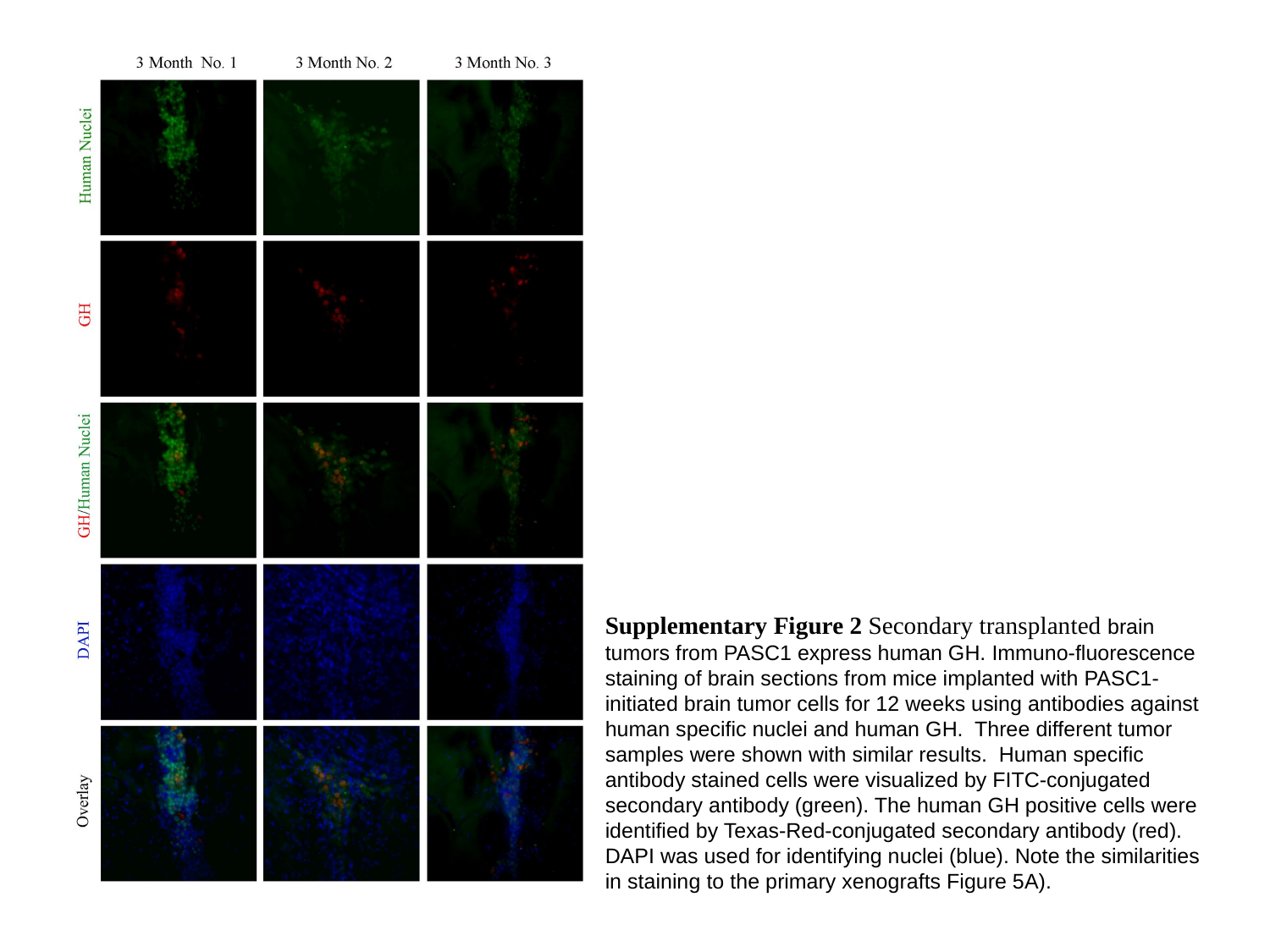

Supplementary Figure 2 Secondary transplanted brain tumors from PASC1 express human GH. Immuno-fluorescence staining of brain sections from mice implanted with PASC1-initiated brain tumor cells for 12 weeks using antibodies against human specific nuclei and human GH. Three different tumor samples were shown with similar results. Human specific antibody stained cells were visualized by FITC-conjugated secondary antibody (green). The human GH positive cells were identified by Texas-Red-conjugated secondary antibody (red). DAPI was used for identifying nuclei (blue). Note the similarities in staining to the primary xenografts Figure 5A).
