# Supplementary material for: Isolation of tumour stem-like cells from benign tumours
Source: Br J Cancer. 2009 Jun 30;101(2):303–11. doi: 10.1038/sj.bjc.6605142 (PMC2720199; doi:10.1038/sj.bjc.6605142)
Supplement: Supplementary Table S1 [file 6605142x3.doc]

**Supplementary Table 1** **Oligonucleotide sequences used in real-time PCR analysis**.

| Gene | Forward | Reverse |
| --- | --- | --- |
| GAPDH | CGTCTTCACCACCATGGAGA | CGGCCATCACGCCACAGTTT |
| CD133 | 5’-GCATTGGCATCTTCTATGGTT-3’ | 5’-CGCCTTGTCCTTGGTAGTGT-3’ |
| MSI1 | 5'-GAGACTGACGCGCCCCAGCC-3' | 5'-CGCCTGGTCCATGAAAGTGACG-3' |
| CD90 | 5’-CGCTCTCCTGCTAACAGTCTT-3’ | 5’-CAGGCTGAACTCGTACTGGA-3’ |
| OCT4 | 5’-CCTGAAGCAGAAGAGGATCA-3’ | 5’-CCGCAGCTTACACATGTTCT-3’ |
| Notch-4 | GCGGAG GCAGGGTCTCAACGGATG | AGGAGGCGGGATCGGAATGT |
| Jagged-2 | ACCAGGTGGACGGCTTTG | CCGCGACAGTCGTTGA; |
| DLL-1 | CCTACTGCACAGAGCCGATCT | ACAGCCTGGATAGCGGATACAC |
| cIAP1 | 5′-CAGCCTGAGCAGCTTGCAA-3′ | 5′-CAAGCCACCATCACAACAAAA-3′ |
| NAIP | 5′-GCTTCACAGCGCATCGAA-3′ | 5′-GCTGGGCGGATGCTTTC-3′ |
| XIAP | 5′-AGTGGTAGTCCTGTTTCAGCATCA-3′ | 5′-CCGCACGGTATCTCCTTCA-3′ |
| BCL-2 | 5′-CATGCTGGGGCCGTACAG-3 | 5′-GAACCGGCACCTGCACAC-3′ |
| MDR-1 | 5'-TGCTCAGACAGGATGTGAGTTG-3’ | 5'-TAGCCCCTTTAACTTGAGCAGC-3' |
| MRP-3 | 5'-CTTAAGACTTCCCCTCAACATGC-3' | 5'-GGTCAAGTTCCTCTTGGCTC-3' |
| MRP-1 | 5'-CAATGCTGTGATGGCGATG-3’ | 5'-GATCCGATTGTCTTTGCTCTT-3' |
| BCRP-1 | 5'-TGGCTGTCATGGCTTCAGTA-3' | 5'-GCCACGTGATTCTTCCACAA-3' |
| LH | 5’-GCCATCCTGGCTGTCGAGAAG–3’ | 5’-GAGCCGGATGGACTCGAAGCG–3’ |
| TSH | 5’-ACAATGCACATCGAAAGGAGA–3’ | 5’-TCCTGGTATTTCTACAGTCCT–3’ |
| FSH | 5’-ATAGAGAAAGAAGAATGTCGT–3’ | 5’-GTGAGCACAGCCGGGCACTCT–3’ |
| ACTH | 5’-AGCTTGGCCATATCTGATATG–3’ | 5’-GATGTAGCGGTCCGCAGCAAT–3’ |
| GH | 5’-ATGACACCTATCAGGAGTTTGAAGAAG-3’ | 5’-GATGCGGAGCAGCTCTAGGTTAGATTT-3’ |
| PRL | 5’-GGGTTCATTACCAAGGCCATCA-3’ | 5’-TTCAGGATGAACCTGGCTGAC-3’ |
| PROP1 | 5’-GAGTCAGCCTTTGGGAGGAAC–3’ | 5’-TGGTGGTGGTGGTGCTGCGTA–3’ |
| Pit1 | 5’-ACAGCTGCTGATTTCAAGCA-3’ | 5’-ACAAAGCTCCTACTTGCTCA-3’ |
| Alpha-subunit | 5’-TCCGCTCCTGATGTGCAGGAT-3’ | 5’-GGACCTTAGTGGAGTGGGATA–3’ |
